# Measurements of left ventricular myocardial longitudinal systolic displacement using spectral and colour tissue Doppler: time for a reassessment?

**DOI:** 10.1186/1476-7120-7-12

**Published:** 2009-03-17

**Authors:** Aristomenis Manouras, Kambiz Shahgaldi, Reidar Winter, Lars-Åke Brodin, Jacek Nowak

**Affiliations:** 1 Department of Clinical Physiology, Karolinska University Hospital in Huddinge, Stockholm, Sweden; 2 Department of Cardiology, Karolinska University Hospital in Huddinge, Stockholm, Sweden; 3 School for Technology and Health, Royal Institute of Technology, Flemingsberg, Stockholm, Sweden

## Abstract

**Background:**

Echocardiographic measurements of left ventricular (LV) myocardial displacement may produce different results depending on the choice of employed modality and subjective adjustments during data acquisition and analysis.

**Methods:**

In this study, left ventricular longitudinal systolic displacement was quantified in 57 patients (31 women and 26 men, 50 ± 16 years) using colour (colour TD) and spectral tissue Doppler (spectral TD) before and after temporal filtering (30 to 70 milliseconds in 20-millisecond steps) and changed offline gain saturation (0%, 50% and 100%), respectively. The results were compared with those obtained with anatomic M-mode.

**Results:**

Whereas only minor differences occurred between the results of colour TD and anatomic M-mode measurements, spectral TD significantly overestimated the results obtained with both these methods. However, the limits of agreement between the results produced by all three studied methods were not clinically acceptable in any of the cases. The spectral TD displacement values increased along with increasing offline gain saturation whereas the effect of temporal filtering on colour Doppler measurements was insignificant.

**Conclusion:**

Measurements of LV myocardial longitudinal displacement employing spectral TD, colour TD or anatomic M-mode produce different results, thus discouraging interchangeable use of these modalities. Whereas the results of spectral TD measurements can be significantly altered by changing offline gain setting, the effect of temporal filtering on colour TD measurements is insignificant, a fact that increases clinical practicality of the latter method.

## Background

Left ventricular longitudinal systolic myocardial shortening is an important component of the wringing myocardial motion generated by contractile activity of helically oriented subepicaridial and subendocardial fibers [1-3]. As a result, the mitral annulus moves toward the apex that itself remains relatively stationary [4-8]. The systolic atrioventricular plane displacement was shown to correlate well to global LV function and its direct measurement, performed originally with M-mode based methodology, soon became well established [9-13]. Further refinement of the conventional M-mode approach in the measurement of the amplitude of systolic myocardial displacement includes anatomic M-mode that enables angle corrected alignment of the M-mode ultrasound beam [14,15].

The adaptation of pulsed Doppler signal to measurements of low velocity myocardial movements laid the foundations for the diagnostic use of spectral TD method [15] that, unlike M-mode modalities, allows assessment of systolic myocardial shortening by temporal integration of the recorded systolic myocardial velocity curves from the basal segments of LV wall. In recent years, spectral TD has become a highly appreciated tool in the evaluation of myocardial contractility being broadly employed in clinical research and practise for the assessment of LV function. Another increasingly successful approach to myocardial velocity imaging has been created by the development of 2-dimensional imaging of myocardial motion velocities as colour-coded maps [16,17], and the modern colour tissue Doppler modality provides detailed information about myocardial longitudinal velocity and displacement at practically any discrete point within the myocardial wall.

During the years following their introduction, M-mode based measurement of the amplitude of systolic myocardial displacement and the measurements of myocardial motion velocities with spectral and colour TD have been extensively used for the echocardiographic evaluation of myocardial function. Especially the colour TD modality that has been subject to remarkably rapid technical and conceptual refinements is now becoming dominating echocardiographic method in diagnostic cardiology. However, although a good correlation has been found between the results generated by M-mode, spectral and colour TD methods, there has been a growing awareness that the results obtained with these methods may not agree in a clinically acceptable way [18-21]. In addition, not even modern echocardiographic equipment can provide ideal, noise-free signal, a fact that may create a need of signal processing using temporal filters or gain adjustment. In turn, these subjective adjustments during echocardiographic data acquisition and analysis may have a significant impact on the results of the performed measurements and thereby also on the observed differences between the used methods. We found it therefore of interest to compare spectral and colour TD measurements of LV myocardial longitudinal systolic displacement with direct measurements of myocardial displacement using colour-coded anatomic M-mode, with special reference to the impact of temporal filtering and different offline gain setting on the results produced by the respective Doppler based methods

## Methods

The study population included 57 patients consecutive (51 ± 16 years) referred to transthoracic echocardiography on clinical grounds. They were all in sinus rhythm and were examined with both conventional echocardiography and with tissue Doppler imaging. All echocardiographic recordings were performed by the same experienced sonographer. The study was approved by the ethics committee at Karolinska University Hospital, Stockholm Sweden.

### Conventional echocardiography

The echocardiographic examinations were performed according to the guidelines of American Society of Echocardiography [22] using commercially available Vivid 7 equipment (GE Vingmed, Horten, Norway) and a standard phased array 2.5 MHz multi-frequency transducer. All images were acquired in second harmonic mode at the end of expiration from parasternal, as well as from apical 2- and 4-chamber views with the patients in left lateral position. ECG was recorded simultaneously.

### Tissue velocity echocardiography

#### 1. Colour TD

Cineloops of three to six consecutive heartbeats were acquired in each subject with a high temporal resolution (19 cineloops with 100 frames/s, 22 cineloops with 104 – 105 frames/s, 15 cineloops with 154 – 162 frames/s, and 1 cineloop with 184 frames/s). Off-line analysis of digitally stored formatted raw data containing grey scale and colour Doppler tissue velocity information was performed using Echopac software (Echopac, version 6.0.0, GE Vingmed Ultrasound, Norway).

**Figure 1:**
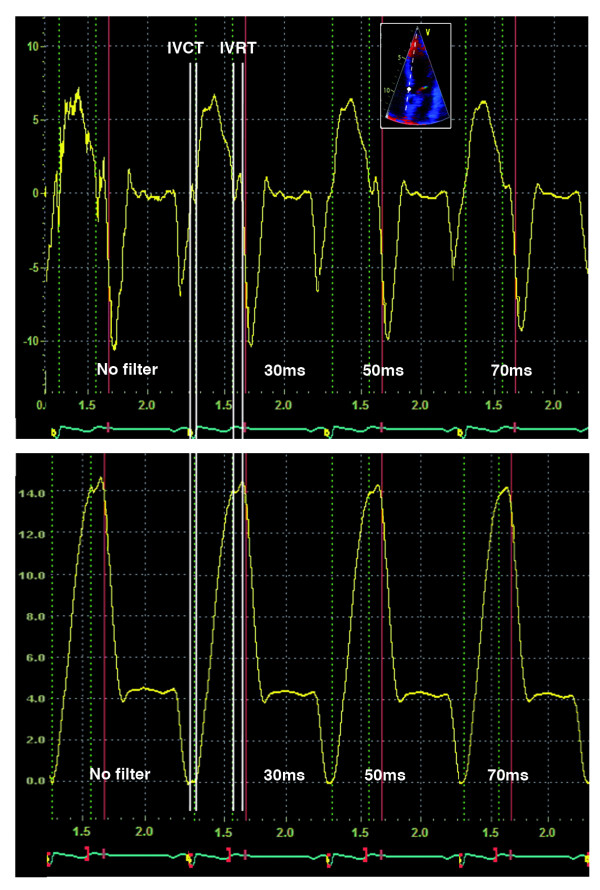
**Typical LV longitudinal myocardial velocity curves obtained with colour TD at different temporal filter widths (*top*) from the most basal segment of septum (*insertion*)**. Integration of the velocity curves over time yielded the corresponding longitudinal myocardial displacement curves (*bottom*). Isovolumic contraction time (IVCT) and isovolumic relaxation time (IVRT) as well as the applied temporal filters are indicated.

**Figure 2:**
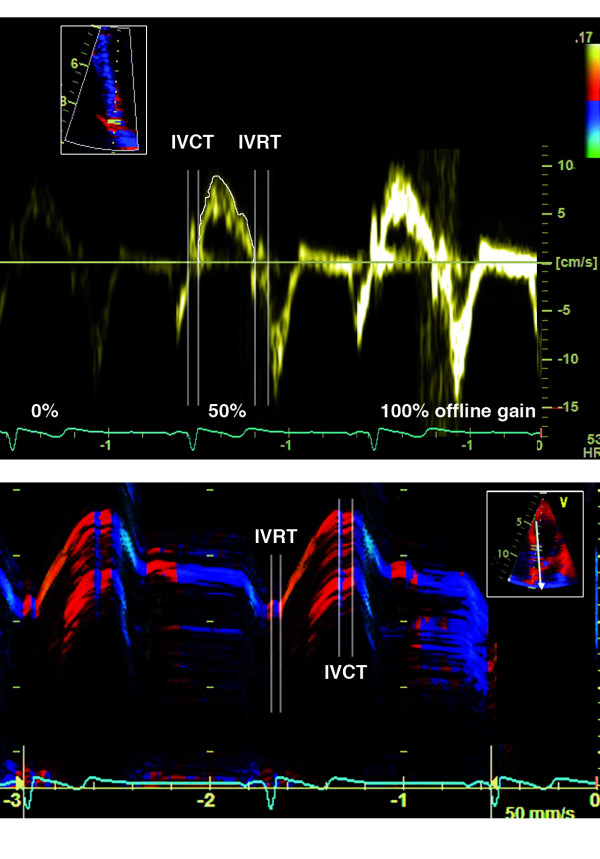
**(*Top*) Typical LV longitudinal myocardial velocity curves obtained with spectral TD at different offline gain settings from the most basal segment of septum (*insertion*)**. The respective curves were traced at the outer border of the velocity waveform as indicated and integrated over time to yield the corresponding longitudinal myocardial displacement values. Isovolumic contraction (IVCT) and relaxation (IVRT) periods as well as the chosen offline gain saturation are indicated. (*Bottom*) Typical colour-coded anatomic M-mode image of LV longitudinal myocardial motion acquired at basal septum (*insertion*) used for the measurements of systolic displacement. Boundaries of isovolumic contraction (IVCT) and relaxation (IVRT) periods were established by observing the specific colour bands expressing the reversal of velocity direction at the beginning and the end of systole.[23]

The employed software allows real-time digital acquisition of tissue velocity curve at any site in the myocardium in the stored cineloops and the obtained velocity curves can subsequently be integrated over time giving the corresponding myocardial displacement curves (Figure 1). The tissue displacement analysis was performed from the optimal (depending on image characteristics) measuring point using 5 × 5 pixels sampling volume set at the junction between the respective basal segment of septal, lateral, anterior and inferior LV walls and the corresponding sector of mitral annulus. Colour TD-derived myocardial displacement during the systolic ejection was established by measuring the amplitude of the myocardial displacement curve during LV ejection after exclusion of isovolumic contraction and relaxation phases. Delineation of the respective isovolumic periods was established by defining the position of their respective starting and ending points on the myocardial velocity curve as described earlier [23]. Accordingly, the end of the isovolumic contraction period was set at the zero-crossing point for the ascending limb of the myocardial tissue velocity curve at the beginning of the systolic ejection, and the beginning of the isovolumic relaxation period was defined by the zero-crossing point for the descending limb of the myocardial tissue velocity curve at the end of systolic ejection (Figure 1, *top*). These events coincided with the specific colour bands in the corresponding colour-coded anatomic M-mode images [23] (Figure 2, *bottom*). In one of the patients, in whom a clear zero-crossing point could not be detected, the end of the isovolumic contraction period was set at the beginning of the ascending limb of the velocity curve at the start of the systolic ejection. The beginning of the isovolumic relaxation phase was in this case set at the end of the descending limb of the myocardial velocity curve following the systolic ejection, immediately before the start of positive wave during the isovolumic relaxation.

The myocardial velocity data for the calculation of myocardial displacement were acquired initially without any temporal filtering. While keeping the data sampling point unaltered, the selected cineloops were then subjected to gradual filtering using 30, 50 and finally 70 milliseconds filters. All measurements were performed on 3 cardiac cycles and the results were averaged.

#### 2. Spectral TD

Spectral TD velocities were acquired from an optimal measuring site at the junction between the respective basal segment of septal, lateral, anterior and inferior LV wall and the corresponding sector of mitral annulus using 5 mm sampling volume. Care was taken to keep the data sampling point placed continuously on the ventricular myocardium and the angle of incidence as parallel as possible to the axis of the longitudinal myocardial movements throughout the entire cardiac cycle. At least 3 consecutive cardiac cycles were recorded. Nyquist's limit was adjusted during each recording. Standard gain settings were used with a receive gain of 0 dB. The raw data containing both grey-scale and TD information was stored digitally for subsequent offline analysis on a computer equipped with Echopac software (Version 6.0.0, GE Vingmed Ultrasound, Norway).

The spectral TD signal from 3 cardiac cycles was traced at the envelope of the velocity waveform (Figure 2, *top*) and the velocity time integral expressing systolic myocardial displacement was measured and averaged. The isovolumic displacement was excluded. The starting and ending point of the respective isovolumic periods was defined using the same procedure as for colour TD. The initial measurements were carried out with the manufacturer's default offline gain setting, i.e. 50% saturation of the spectral signal. All measurements were then repeated using desaturated (0% saturation – spectral signal barely visible) and fully saturated (100% saturation) offline gain.

### Anatomic M-mode echocardiography

Anatomic M-mode modality is available with the Echopac software and allows free positioning of M-mode line at any site in the 2-D myocardial image [14,15]. Anatomic M-mode traces from the basal septal, lateral, anterior and inferior segment of the myocardium at the junction to the respective sector of the mitral annulus were obtained by positioning the cursor parallel to the axis of the longitudinal LV motion (Figure 2). The correction angle was in all cases less then 30 degrees. By superimposition of colour TD on grey-scale 2D-image, the changes in the direction of myocardial displacement in the selected M-mode line at the beginning and the end of the isovolumic phases could readily be identified [23] (Figure 2, *bottom*) and the respective isovolumic periods were then excluded from the measurements. The measurements of myocardial displacement were performed in each above-mentioned LV segment on two cardiac cycles and were then averaged.

### Statistical analysis

In order to assess the reproducibility of colour-coded and spectral TD measurements, a second independent observer with similar echocardiographic experience performed a second measurement of colour-coded, spectral TD and anatomic M-mode displacements. Measurements were performed at basal inferior LV segment in 20 patients. Reproducibility was expressed as methodological error (E) in a single measurement estimated from double measurements according to the formula E = [SD of the difference/(total mean * √2)] * 100%.

All data are presented as mean ± standard deviation (SD). The statistical significance level was set at p < 0.05. Group comparisons of continuous variables were performed using analysis of variance (ANOVA), with subsequent multiple comparisons with the use of Scheffé's test.

Univariate relations between the results of the evaluated TD methods as well as their relation to the results of anatomic mode were tested with standard regression analysis. Student's t-test was used when suitable for comparisons of paired data. The statistical analyses were carried out using standard statistical software (SPSS version 11.01). Assessment of the agreement between the results the evaluated methods was performed according to Bland Altman [24].

## Results

Additional file 1 provides demographic and clinical characteristics of the studied population. As can be seen, there was no significant difference in age distribution in the two gender subgroups. BSA was significant lower in women (p < 0.001), but LV mass and LV ejection fraction was not significant different. The number of different pathologies was somewhat higher in women.

The error of the double measurements of LV displacement performed by two independent observers was 6.9% with unfiltered colour TD, 7.4% with colour-coded anatomic M-mode, and 13.6% with spectral TD at 50% offline gain saturation.

With the default offline gain setting of 50% saturation, the results of LV myocardial displacement measurements by spectral TD correlated well with those obtained using anatomic M-mode (R = 0.80; p < 0.001) whereas a stronger correlation was observed between anatomic M-mode and unfiltered colour TD measurements (R = 0.95; p < 0.001). Furthermore, there was a significant correlation between the results of spectral and colour TD measurements (R= 0.83; p < 0.001) as well. However, despite the significant relationships between the displacement values measured with the three tested methods, the obtained results differed. The LV longitudinal systolic displacement values obtained with spectral TD were significantly higher (p < 0.001) than those measured with anatomic M-mode and the difference increased at increasing offline gain saturation [see Additional file 2]. On the other hand, the difference between the results of colour TD and anatomic M-mode was smaller, even if it was statistically significant as well. Along with increasing temporal filtering the difference decreased further, predominantly in septal and inferior LV wall, whereas smaller increase was observed in lateral and anterior wall.

**Figure 3:**
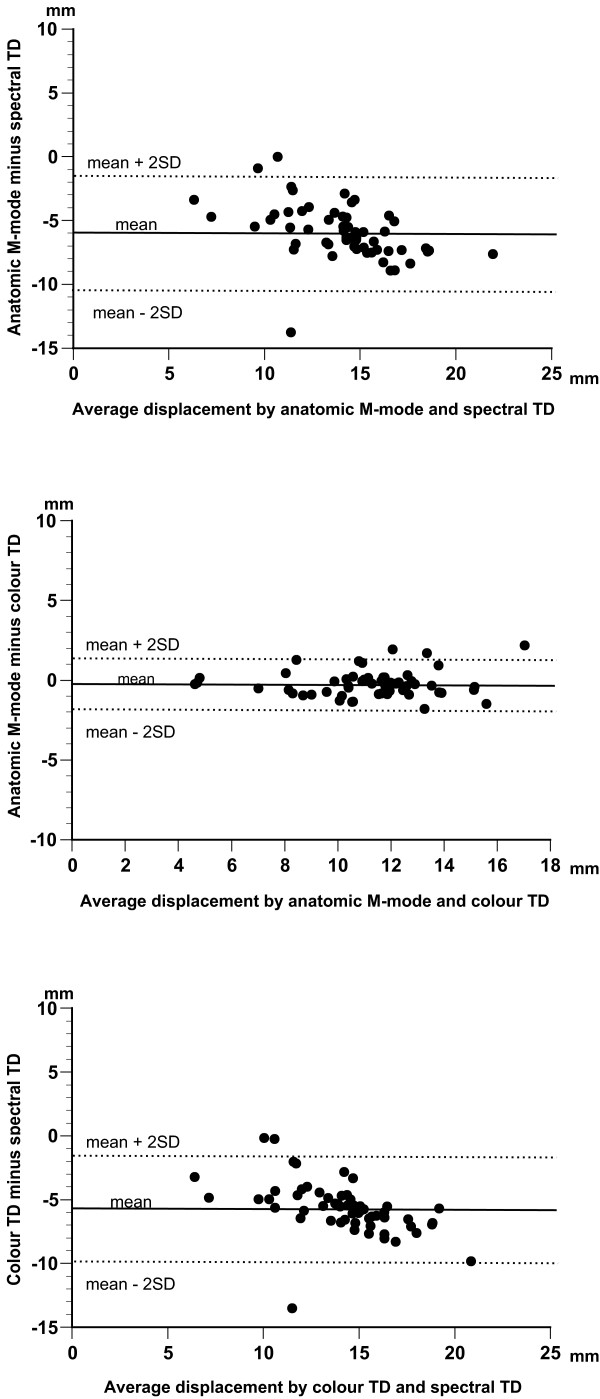
**Bland-Altman plot of differences between LV myocardial longitudinal systolic displacement measured by anatomic M-mode and spectral TD (*top*), anatomic M-mode and colour TD (*middle*), and by colour and spectral TD (*bottom*)**.

The poor agreement between the outcomes of spectral TD and anatomic M-mode is evident from the results of Bland Altman analysis. As can be seen, spectral TD method overestimated M-mode measurements by a mean value of at least 5.5 mm with a considerable possible variation as reflected by rather wide limits of agreement for the measured variables [see Additional file 3], Figure 3, *top*). On the other hand, the mean difference between the results of colour TD and anatomic M-mode did no exceed 1.0 mm and the limits of agreement were much narrower ([see Additional file 3], Figure 3, *middle*) implying better agreement between the results obtained with these two methods. Consistent with the results mentioned above, the agreement between the results of colour and spectral TD measurements was poor, with a constantly higher (by a mean value of at least 4.8 mm) displacement values for spectral TD and rather wide limits of agreement ([see Additional file 3], Figure 3, *bottom*).

**Figure 4:**
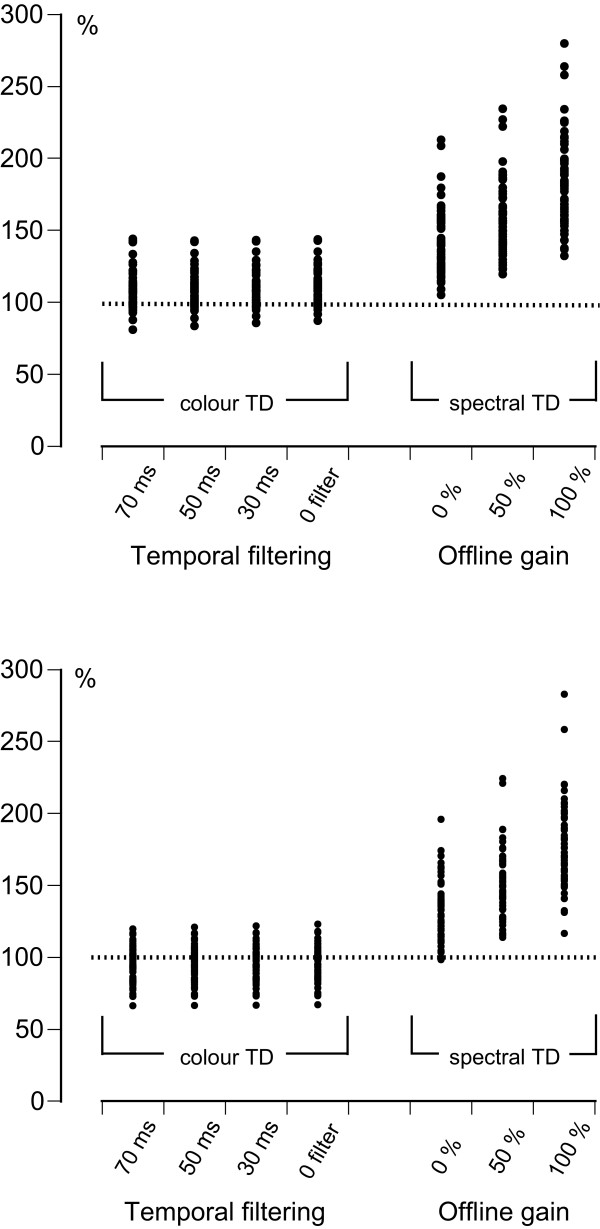
**Distribution of LV myocardial longitudinal systolic displacement values obtained at the basal segment of septal (*top*) and lateral LV wall (*bottom*) with colour TD at different filter widths and with spectral TD at different offline gain levels**. The individual data points are expressed as a percentage of the corresponding individual anatomic M-mode values that are considered to represent a value of 100%.

The distribution of the LV myocardial longitudinal systolic displacement values measured in the basal septal and lateral segments with colour and spectral TD at different filter widths or offline gain settings is presented in Figure 4. As can be seen from the figure, changing spectral TD gain setting from unsaturated to fully saturated gain resulted in a clear alteration of the measured displacement, with increasing values at increasing gain saturation and consequently, increasing difference between spectral TD and anatomic M-mode measurements. On the other hand, the impact of increasing temporal filtering on the colour TD derived displacement was barely discernible, with a slight tendency to lower displacement values at increasing filter width. As can be seen from Additional file 4, a mean difference between the results of unfiltered and maximally filtered (70 ms) colour TD measurements was not greater than 0.2 mm and the widest limits of agreement amounted to ± 0.6 mm, thus implying that the effect of temporal filtering lacked any clinical significance. On the other hand, the results of spectral TD measurements using desaturated and fully saturated offline gain differed by a mean value of at least 4.1 mm with the widest limits of agreement of ± 5.6 mm indicating a significant effect of the choice of offline gain setting.

## Discussion

The currently observed differences between the results provided by the three different echocardiographic modalities is not entirely surprising since the methods represent three independent approaches to the assessment of systolic LV displacement. Both spectral and colour TD method provides quantification of regional myocardial velocities as a base for a subsequent calculation of myocardial displacement. However, different methodologies are used for the computation of velocity curves. Spectral TD detects frequency shift between emitted and returning ultrasound beam and the acquired data are then analysed using Fast Fourier Transform technique [25]. The obtained velocity wave contains a spectrum of velocities from the interrogated myocardial location and the maximal velocity can be measured at the envelope of the velocity curve. Different from spectral TD, colour TD estimates frequency shift indirectly using autocorrelation analysis and provides instead average of all velocities within the myocardial region of interest [25]. Hence, the maximal velocity values obtained with colour TD should be expected to be lower than those extracted from spectral TD velocity wave.

In contrast to spectral and colour TD, anatomic M-mode focuses on the direct measurement of the amplitude of the myocardial motion during cardiac cycle by analysis of 2D samples in time domain along freely positioned virtual M-mode line and the information is then displayed in a way similar to that of conventional M-mode [14,15]. Measurement of LV dimensions and atrioventricular plane movements during systole using conventional M-mode has been found to be an accurate method for the assessment of regional and global left ventricular function [9-11,26] The anatomic M-mode technique further increases accuracy of the M-mode measurements by reducing possible errors produced by inappropriate alignment of M-mode ultrasound beam non-orthogonal to the interrogated myocardial segment [14]. Overestimation of the systolic LV shortening, especially in the lateral atrioventricular margin where the measured displacement would represent a mean vector of the longitudinal and the concomitant radial LV motion, can thus be avoided. The accuracy of anatomic M-mode has been found to increase with second harmonics imaging, but to decrease at correction angles > 60° [15]. Therefore, in order to ensure optimal accuracy of the performed measurements, all images in the present study were acquired in second harmonics mode, and the correction angle never exceeded 30°.

When considering the results of the current comparison between the three echocardiographic modalities, it should be kept in mind that for technical reasons, the measurements with spectral TD could not be performed on the same cardiac cycles as those chosen for the colour TD and colour-coded anatomic M-mode measurements. Although the spectral and colour TD imaging followed each other closely and no significant heart rate alterations occurred between the respective registrations, a possible influence of the separate image acquisition on the relationship between the measured variables still cannot be excluded. In addition, both spectral and colour TD measurements are prone to be affected by alterations of insonation angle and the influence of such variations on the obtained results cannot be entirely excluded either. However, all image acquisition in the present study was performed by the same experienced sonographer and a special care was taken to keep the Doppler angle parallel with the LV long axis, with the sampling volume at the same location within the myocardium. Such a procedure would certainly minimize any possible effects caused by random alterations of insonation angle. Furthermore, it appears unlikely that any of the above-mentioned confounding factors would act exclusively in one direction, and the internal consistency of the present results showing clear differences between the evaluated modalities contest any significant blurring effect of these factors.

The present results reveal the occurrence of significant differences between the results of displacement measurements obtained with the three employed echocardiographic methods. Although the mean (all four LV walls) displacement values obtained with colour TD was statistically significantly higher than the corresponding values produced by anatomic M-mode, the magnitude of the difference seemed to be clinically negligible. The constant difference between the anatomic M-mode and colour TD mean displacement values was only -0.2 ± 0.8 mm. However, these figures also imply that the displacement measurements by the two methods may differ by as much as -1.8 to +1.4 mm. In view of the current interobserver variability of anatomic M-mode (7.4%) and colour TD (6.9%) that would produce variability of the respective results by not more than ± 0.8 mm, these limits of agreement, even if fairly narrow, can hardly be clinically accepted. In addition, the limits of agreement for the anatomic M-mode and colour TD measurements in individual LV walls were even wider [see Additional file 3].

The current results are in keeping with those reported from the study of Ballo et al. [21] in which spectral TD overestimated the displacement values obtained with the conventional M-mode method employed by these authors to the same degree as it was found in the present experiments. At the same time, the authors reported greater than that currently observed overestimation by M-mode of the results produced by colour TD method. However, the measurements in the above-mentioned study [21] were performed exclusively in lateral LV wall and it is often difficult to achieve an appropriate angle of incidence in this LV region. Consequently, the magnitude of the observed difference between conventional M-mode and colour TD modalities might to some extent reflect possible angle-dependent overestimation of the true LV ejectional shortening by M-mode. Similar results were reported from the study of Yumi Hayashi et al. [27], in which the colour TD displacement was generally slightly lower than the displacement measured with anatomic M-mode. The small discrepancies between the present results and those reported by Yumi Hayashi et al. [27] may be caused by the fact that, contrary to the present measurements, the displacement values in the above-mentioned study also included isovolumic myocardial movements.

The currently observed overestimation of the results of anatomic M-mode and colour TD measurements by spectral TD was not only statistically but also clinically significant. The present results are in keeping with the previous reports of significantly higher systolic velocity values measured by spectral TD than by colour TD modality [18,19] and demonstrate that spectral TD displacement values are higher than those provided by direct displacement measurements with anatomic M-mode as well. In addition, reproducibility of spectral TD measurements of myocardial motion velocities has been demonstrated to be low [28], and even if the process of temporal integration of velocity curves for calculation of myocardial displacement has by itself a smoothing effect, both the dispersion of the current individual spectral TD displacement values as well as the interobserver variability was clearly higher than the respective values for the two other methods. Consequently, the agreement between spectral TD and these methods was poorer than what was observed with colour TD and anatomic M-mode. The constant differences between spectral TD and the two other modalities amounting to almost -6 mm and the wide limits of agreement with anatomic M-mode (at least -9.6 to -2.0 mm or wider) and colour TD (at least -8.4 to -1.2 mm or wider) cannot be clinically accepted and discourage the use of spectral TD for the measurements of myocardial displacement interchangeably with the two other methods.

The present study demonstrates that beside the choice of measuring modality, also adjustments made by sonographer during echocardiographic data acquisition and analysis can significantly influence the accuracy of the myocardial displacement measurements. In fact, Lui et al. identified the Doppler gain as one of the most significant sources of error and variability in *in vitro *model of pulsatile flow [29]. Increasing gain would result in increasing spectral broadening and upward shift of the upper border of the spectral velocity curve, with higher measured velocity values as a consequence. Not surprisingly then, the effect of offline gain saturation on the results of the present spectral TD measurements was indeed highly significant. Furthermore, it is important to emphasize in this context that the magnitude of the currently observed gain saturation effect on the spectral TD displacement, both in terms of measured values and their dispersion, is of crucial clinical relevance since the measurements with unsaturated and fully saturated offline gain lacked any clinically acceptable agreement at all [see Additional file 4]. In contrast, the effect of temporal filtering on the present colour TD measurements was barely measurable and clinically negligible, thus rendering this modality more robust in the clinical setting.

Measurement of LV myocardial longitudinal motion with spectral and colour tissue Doppler technique is now a generally accepted procedure for the assessment of global and regional LV function [30]. Together with the conventional M-mode still broadly used for this reason, the two Doppler based methodological approaches have been hitherto often used interchangeably. However, these technologies have entered clinical diagnostic laboratories long before their impact was fully recognised and there are still only limited data on their effectiveness in population screening in the high volume echocardiographic laboratories. In fact, the effectiveness of colour TD in the detection of coronary artery disease has been addressed in some large-scale studies [31,32] but similar studies addressing spectral TD remain still to be performed. Against this background, the present results have important clinical implications since they reveal the occurrence of systematic differences between the results produced by different tissue Doppler and M-mode based methods and highlight a need of reassessment of the diagnostic qualities of the Doppler modalities. The currently observed differences between the three evaluated echocardiographic modalities and the different degree of sensitivity of the tissue Doppler methods to offline processing do not support their interchangeable use and emphasize the need of specific for each method normal values when comparing spectral, colour and anatomic M-mode measurements. Furthermore, the observed high sensitivity of spectral TD to offline gain setting calls for strict standardization of the offline data processing when using this method, or alternatively, the use of gain level specific normal values. This, together with the recently reported low reproducibility of spectral TD velocity measurements raises, however, serious doubts about the applicability of this modality in the routine echocardiographic practice.

## Conclusion

Measurements of LV myocardial longitudinal systolic displacement with spectral TD, colour TD and anatomic M-mode modalities produce significantly correlated, but different results. Whereas there appears to occur only a minor difference between colour TD and anatomic M-mode measurements, the spectral TD method significantly overestimates the displacement values obtained with both these modalities. However, the limits of agreement between the results generated by all three evaluated methods cannot be clinically accepted in any of the cases and the respective modalities should not be used interchangeably. The outcomes of spectral TD measurements can be significantly altered by varying offline gain setting whereas the effect of temporal filtering on colour TD measurements is insignificant, undoubtedly an advantage when using this modality in clinical practice.

## Competing interests

The authors declare that they have no competing interests.

## Authors' contributions

AR contributed to the design of the study, performed measurements and calculations from ultrasound data as well as statistical analyses, and participated in the interpretation of the results and preparation of the manuscript. KS participated in data collection and interpretation of the results. RW and LÅB supervised the study and contributed to the interpretation of the obtained results. JN supervised the study, contributed to the analysis and interpretation of the data, and was responsible for the preparation of the final version of the manuscript. All authors read and approved the final manuscript.

## Supplementary Material

Additional file 1 **Table S1.** Clinical characteristics of the studied population. Demographics and clinical data on the population studied Click here for file

Additional file 2 **Table S2. **LV myocardial longitudinal systolic displacement measured with anatomic M-mode, and with colour and spectral TD using different filter and gain settings. The data presented provide myocardial systolic longitudinal displacement values measured with 3 different echo modalities, i.e. anatomic M-mode, colour TD and spectral TD. Click here for file

Additional file 3 **Table S3.** Mean differences and limits of agreement (mean ± 2SD) for LV myocardial longitudinal systolic displacement values obtained with anatomic M-mode, colour TD without temporal filtering, and spectral TD with 50% offline gain saturation. The data presented give mean values and limits of agreement for myocardial systolic displacement measured with 3 different echo modalities, i.e. anatomic M-mode, colour TD and spectral TD. Click here for file

Additional file 4 **Table S4.** Mean differences and limits of agreement (mean ± 2SD) for LV myocardial longitudinal systolic displacement values obtained with unfiltered and maximally filtered colour TD, and with minimally and maximally gain saturated spectral TD. The data presented provide mean values and limits of agreement for myocardial systolic displacement measured with temporally filtered colour TD and offline gain modulated spectral TD. Click here for file
